# Myocardial Bridging and Cardiac Arrest

**DOI:** 10.1016/j.jaccas.2025.106736

**Published:** 2026-01-27

**Authors:** Karthik V. Iyer, Robert J. Hieger, Rahul Rajput, Katherine L. Raymer, Ananya P. Ganesan, Souheil Khoukaz, Bassam Roukoz, Bassem Mikhail, Jooho P. Kim, Vikram V. Oke

**Affiliations:** a Department of Internal Medicine, Mercy Hospital Jefferson, Festus, Missouri, USA; b Department of Family Medicine, Mercy Jefferson Hospital, Festus, Missouri, USA; c Lake Erie College of Osteopathic Medicine, Seton Hill, Greensburg, Pennsylvania, USA; d Department of Cardiology, Mercy Hospital Jefferson, Festus, Missouri, USA; e Department of Pulmonology, Mercy Hospital Jefferson, Festus, Missouri, USA

**Keywords:** cardiac arrest, coronary anomaly, left anterior descending artery, myocardial bridging, ventricular fibrillation

## Abstract

**Background:**

Myocardial bridging (MB) is a congenital coronary anomaly in which a segment of an epicardial artery tunnels through the myocardium. Although frequently considered benign, MB has been increasingly linked to ischemia, malignant arrhythmias, and sudden cardiac death.

**Case Summary:**

We describe a case of a 39-year-old woman with mid–left anterior descending artery MB who presented with sudden chest pain followed by cardiac arrest. Coronary angiography revealed MB without obstructive disease. Despite prolonged resuscitation and hypoxic brain injury, she achieved functional recovery after intensive care.

**Discussion:**

We compared our patient with 10 previously published cases of MB-associated cardiac arrest. Patients were typically men with ventricular fibrillation as the universal arrest rhythm, and the left anterior descending artery was most commonly involved, with variable outcomes.

**Take-Home Messages:**

MB should not be regarded as uniformly benign. Recognition of its malignant potential—particularly in patients with no obstructive coronary disease—is critical for diagnosis, management, and secondary prevention.

## Graphical Abstract

**Figure:**
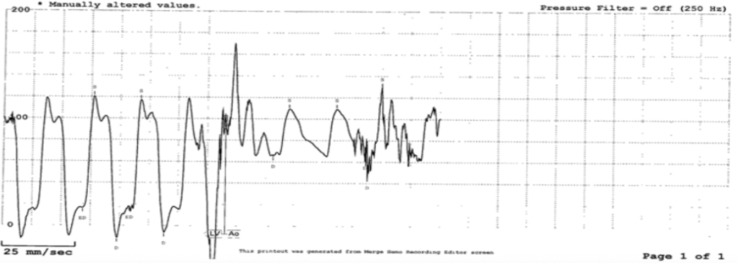

Myocardial bridging (MB) is a congenital coronary anomaly in which a segment of an epicardial artery courses intramyocardially. First described by Reyman in 1732, MB was long regarded as a benign incidental finding.^1^ However, accumulating evidence demonstrates that MB can precipitate angina, myocardial ischemia, malignant arrhythmias, and sudden cardiac death. Prevalence varies by modality, reported in 1.5% to 16% of angiographic studies and up to 80% of autopsy series.^2^^,^^3^

**Take-Home Messages**

• Myocardial bridging should not be regarded as uniformly benign.
• Recognition of its malignant potential—particularly in patients with no obstructive coronary disease—is critical for diagnosis, management, and secondary prevention.

Dynamic compression of the tunneled segment during systole reduces coronary perfusion, particularly in tachycardic states. Repetitive systolic compression creates abnormal shear stress that may injure the endothelium, resulting in endothelial dysfunction and impaired vasomotor reactivity. This predisposes to localized vasospasm and turbulent flow within the bridged segment, further compromising diastolic filling. In addition, recurrent flow acceleration and delayed relaxation can extend into the microcirculation and contribute to microvascular ischemia. These hemodynamic and structural changes are believed to promote arrhythmogenesis through heterogeneous repolarization and the development of myocardial fibrosis. These alterations can produce localized zones of delayed conduction and repolarization dispersion, forming the substrate for ventricular arrhythmias—especially under sympathetic stimulation.^4^ Although most cases are asymptomatic, certain anatomical features (bridge length, depth, and degree of systolic compression) and clinical contexts (exertion and vasospasm) increase the risk of ischemia and arrhythmia.

We present a case of out-of-hospital cardiac arrest attributed to mid–left anterior descending artery (LAD) MB and review the literature to highlight patterns and implications of MB as a cause of cardiac arrest.

## Case Presentation

A 39-year-old woman with hypertension, hyperlipidemia, obesity, obstructive sleep apnea, epilepsy, and known mid-LAD MB presented with sudden severe chest pain at night. Sublingual nitroglycerin provided no relief. Shortly thereafter, she collapsed and was found by emergency medical services in ventricular fibrillation. She underwent 4 rounds of defibrillation and multiple rounds of cardiopulmonary resuscitation, achieving return of spontaneous circulation.

**Figure 1:**
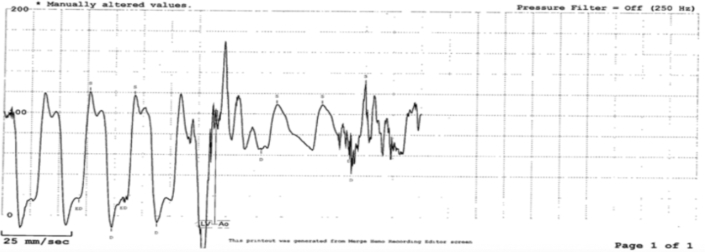
EMS Rhythm Strip EMS rhythm strip showing ventricular tachycardia on arrival, which rapidly deteriorated into ventricular fibrillation requiring defibrillation. EMS = emergency medical service.

**Figure 2:**
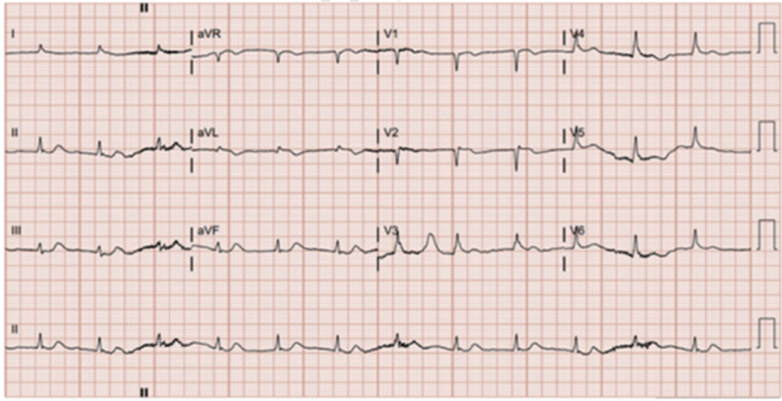
Initial Electrocardiogram Obtained in the Emergency Department Slight ST-segment elevation is present in lead V_2_, consistent with septal myocardial infarction, with a prolonged QRS duration suggesting possible right ventricular conduction delay.

On arrival to the emergency department, the initial electrocardiogram showed ventricular tachycardia evolving into ventricular fibrillation. She had a history of non–ST-segment elevation myocardial infarction with elevated troponin (783 ng/L) and an ejection fraction of 40% to 45%, as shown in Figures 1 and 2.

Cardiac catheterization revealed no obstructive coronary disease but confirmed MB of the mid- to distal LAD. Echocardiography showed mildly reduced global systolic function (ejection fraction 45%-50%) with apical wall hypokinesis (see Video 1 demonstrating MB of the mid- to distal LAD with associated coronary artery spasm).

The patient was admitted to the intensive care unit and treated with targeted temperature management. Her course was complicated by aspiration pneumonia, seizure-like activity, and hypoxic-ischemic brain injury on magnetic resonance imaging. She required tracheostomy and percutaneous endoscopic gastrostomy tube placement but gradually regained neurological function. She was ultimately decannulated and discharged home with functional recovery. Table 1 includes pertinent serum laboratory values obtained upon initial presentation to the hospital’s emergency department.

**Table 1:** Pertinent Laboratory Values at Presentation

Laboratory Test	Patient Value	Reference Range	Units of Measurement
Cardiac biomarkers			
Troponin (baseline)	165	≤10	ng/L
Troponin T (6 h)	783	<11	ng/L
CK-MB	–	–	–
Metabolic/electrolytes			
Sodium (Na^+^)	138	135-145	mmol/L
Potassium (K^+^)	3.7	3.5-5.1	mmol/L
Chloride (Cl^−^)	103	98-107	mmol/L
CO_2_	10	22-29	mmol/L
BUN	17	6-20	mg/dL
Creatinine	1.07	0.51-0.95	mg/dL
Glucose	334	74-99	mg/dL
Total protein	5.8	6.6-8.7	g/dL
Albumin	3.5	4.0-5.0	g/dL
Total bilirubin	0.3	≤1.2	mg/dL
Alkaline phosphatase	98	35-104	U/L
AST	156	<40	U/L
ALT	138	≤33	U/L
GFR	>60	≥60	mL/min/1.73 m^2^
Anion gap	25	5-15	mmol/L
Hematology			
Hemoglobin	13.5	11.9-15.1	g/dL
Hematocrit	40.5	38.0-47.0	%
WBCs	24.6	4.0-11.0	×10^3^ cells/μL
Platelets	335	150-400	×10^3^ cells/μL
Inflammatory/coagulation			
CRP	6.7	≤5.0	mg/L
PT/INR	–	–	–
aPTT	–	–	–
Metabolic/endocrine			
HbA_1c_	4.4	≤5.6	%
Lactic acid	13.9	≤2.0	mmol/L

ALT = alanine transaminase; aPTT = activated partial thromboplastin time; AST = aspartate transaminase; BUN = blood urea nitrogen; CK-MB = creatine kinase-MB; CO_2_ = bicarbonate; CRP = C-reactive protein; GFR = glomerular filtration rate; HbA_1c_ = glycosylated hemoglobin; PT/INR = prothrombin time/international normalized ratio; WBC = white blood cell.

## Discussion

This case adds to a growing body of literature illustrating the malignant potential of MB. Although MB is common and often regarded as a benign congenital anomaly, in certain patients, it can serve as a precipitant of ventricular fibrillation and cardiac arrest. To contextualize our findings, we compared our patient with 10 previously published cases of MB-associated cardiac arrest, as shown in Table 2.

**Table 2:** Clinical Characteristics, Interventions, and Outcomes of Patients With Myocardial Bridging–Related Cardiac Arrest

Patient^a^	Gender	Age, y	Comorbidities	Presentation	Rhythm	Artery	Interventions^b^	Laboratory Results	ECG	MB Dx	Outcome
A	F	50	None	Sudden LOC	VF	Distal LAD and diagonal branches	10 min CPR, 5 defib, intubation 4 d, CCB and ARB	↑ WBC, proBNP, troponin I, D-dimer	RBBB, ST ↑, Q V1-V4	CAG, CMR	Alive Asymp 1 y
B	M	43	Smoked 1 ppd	Sudden LOC	VF	LAD	Defib 5 min, ICD	Normal	T inv anterolateral, normalized 3 d	CAG	Alive Asymp 18 mo
C	M	17	None	Arrest during exercise	VF	Mid-LAD (3 × 1 cm)	3 defib, intubation 48 h, surgical unroofing; postoperatively treated with diltiazem and bisoprolol; consented for ICD insertion due to the history of v-fib	N/A	ST ↓	Coronary CT	Alive Asymp 3 mo
D	M	21	None	Arrest during exercise	VF	LAD (1.8 cm)	CPR, 2 defib	N/A	N/A	Autopsy	Died
E	M	46	LVH, normal stress	Sudden LOC	VF	LCx (1.3 cm)	CPR, defib, debridging operation; no symptoms discharged on CCB	Normal	Negative T waves in the precordial and inferior leads	CAG	Alive Asymp 6 mo
F	F	52	HTN	CP, sudden LOC	VF	Mid-LAD	CPR, defib, antiplatelet agents, high-intensity statins, CCBs; patient remained symptom free on follow-up visits	↑ Troponin I (1.332 ng/mL)	ST ↑ with pathologic Q waves	CAG	Alive Asymp on follow-up
G	M	52	Dilated cardiomyopathy (recipient)	Sudden cardiac death on postop day 2 after heart transplantation	Not specified	Mid-LAD (1.8 cm intramural course)	Heart transplantation (biatrial method) due to the diagnosis of dilated cardiomyopathy	No rejection (C4d negative)	Not specified	Autopsy	Died on postop day 2
H	M	12	Hypoplastic coronaries	CP and LOC during mild exercise	Ventricular arrhythmia	LAD	Loop recorder, advised restriction of exertion	Troponin ↑; stress perfusion CMR: large perfusion defect; MRI: fibrosis	ST ↓	Coronary CT, MRI, CAG	Died Asymp at 1 y follow-up, died shortly after while exercising
I	M	29	Tobacco, alcohol use	First: sudden LOC Second (4 mo later): sudden LOC	First: polymorphic VTach, VF Second: VF	LAD (20 mm, ∼80% systolic compression)	First: CPR, multiple shocks, intubation (“number of days”) Second: multiple rounds of defib, patient refused surgical treatment; discharged on empirical amiodarone	First: pH 7.18, K^+^ 2.8, CPK 8,000 (MB 40) Second: CPK 590 (MB 71)	ST ↑ anterolateral/inferior; later diffuse St-T; +SAECG	CAG	Alive Asymp 2 mo
J	M	17	None	Sudden LOC during exercise	VF	LAD	CPR, defib, ICD implantation	Mild ↑ troponin T; electrolytes and inflammatory markers normal	Transient borderline prolonged QT interval	CAG	Alive Postanoxic encephalopathy with persistent short-term memory loss
K	F	38	HTN, HLD, obesity, OSA, former smoker (quit in January 2024), NSTEMI, epilepsy	CP the day before; CP, SOB, LOC on the day of presentation	VF	LAD	CPR, 3 defib, intubation (15-20 min estimated total time of arrest), tracheostomy and PEG tube placed on day 9 of admission EV-ICD placement, discharged to LTAC with diltiazem	Elevated WBC, troponin T, glucose, AST, ALT, Mg, CRP	Low QRS voltage, possible RV conduction delay, septal MI	CAG	Alive Hypoxemic brain injury

Long-term or guideline-directed medical therapy (eg, β-blockers and anticoagulants) is not included.

↑ = increased; ALT = alanine transaminase; ARB = angiotensin receptor blocker; AST = aspartate transaminase; Asymp = asymptomatic; CAG = coronary angiography; CCB = calcium-channel blocker; CMR = cardiac magnetic resonance; CP = chest pain; CPK = creatine phosphokinase; CPR = cardiopulmonary resuscitation; CRP = C-reactive protein; CT = computed tomography; defib = defibrillation; ECG = electrocardiogram; EV-ICD = epicardial/ventricular implantable cardioverter-defibrillator; F = female; HLD = hyperlipidemia; HTN = hypertension; ICD = implantable cardioverter-defibrillator; LAD = left anterior descending artery; LCx = left circumflex artery; LOC = loss of consciousness; LTAC = long-term acute care facility; LVH = left ventricular hypertrophy; M = male; MB = myocardial bridge; MB Dx = myocardial bridge diagnosis; MI = myocardial infarction; MRI = magnetic resonance imaging; N/A = not available; NSTEMI = non–ST-segment elevation myocardial infarction; OSA = obstructive sleep apnea; proBNP = NT-proBNP 1/4 N-terminal pro–B-type natriuretic peptide; PEG = percutaneous endoscopic gastrostomy; postop = postoperative; ppd = pack-per-day (tobacco use); RBBB = right bundle branch block; RV = right ventricular; +SAECG = positive signal-averaged electrocardiogram; SOB = shortness of breath; ST ↑ = ST-segment elevation; ST ↓ = ST-segment depression; St-T = ST-T segment; T inv = T-wave inversion; VF = ventricular fibrillation; v-fib = ventricular fibrillation; VTach = ventricular tachycardia; WBC = white blood cell.

a Case sources: Patient A = Ki^10^; patient B = Sciahbasi et al^8^; patient C = Toya et al^11^; patient D = Ceauşu et al^7^; patient E = Tio et al^12^; patient F = Oh et al^13^; patient G = Phulware et al ^14^; patient H = Holtrup et al^15^; patient I = Cutler and Wallace^9^; patient J = Knaapen et al^16^; patient K = current case.

b Interventions listed reflect immediate resuscitation measures (eg, CPR, defibrillation, and intubation).

Our analysis of these cases alongside our own reveals several striking consistencies. The mean age was 34 years, with a strong male predominance (73%). The vast majority of patients had no comorbidities, and ventricular fibrillation was the presenting rhythm at the time of cardiac arrest. Most patients collapsed suddenly without prodrome, and nearly one-third experienced arrest during exertion. The LAD was implicated in 82% of cases, aligning with prior reports of MB's anatomic predilection.^5^ Outcomes were heterogeneous: although 73% survived, 27% died, and 2 survivors (including our patient) sustained neurologic sequelae. These findings underscore that MB, although often regarded as benign, carries malignant potential, particularly when involving the LAD, and can lead not only to sudden death but also to long-term morbidity in survivors.

The mechanisms by which MB precipitates malignant arrhythmias remain multifactorial. Dynamic systolic compression of the tunneled artery can impair coronary perfusion, particularly during tachycardia or stress. Because coronary blood flow occurs primarily in diastole, most systolic bridges are clinically silent; however, tachycardia shortens diastolic filling time and can convert an otherwise benign bridge into a clinically significant source of ischemia.^6^ Associated endothelial dysfunction, vasospasm, or myocardial remodeling may further increase arrhythmic risk. Diagnostic challenges arise because MB is frequently discovered incidentally on coronary angiography, and ischemic changes may be absent unless provoked. Advanced imaging, such as intravascular ultrasound, computed tomography angiography, or stress perfusion studies, may help identify high-risk bridges.^1^

Management of MB requires an individualized stepwise approach based on symptom severity, anatomic features, and response to medical therapy. Initial treatment focuses on medical management, primarily with β-blockers or calcium-channel blockers (CCBs) to reduce heart rate, prolong diastole, and lessen systolic compression of the tunneled artery. Some reports suggest CCBs may be superior in cases with associated coronary spasm, as β-blockers can occasionally exacerbate systolic narrowing.^7^ Nitrates should generally be avoided, as they dilate the nonbridged arterial segments, increase the pressure gradient across the bridged area, and reduce preload, leading to reflex tachycardia and worsened compression. Ivabradine may be used off-label in patients intolerant to β-blockers or CCBs, particularly those with reduced ejection fraction.^8^ Patients with mild or incidental findings, such as young athletes, may be managed conservatively with lifestyle modifications—avoiding excessive exertion, stress, and stimulants—while undergoing continued monitoring or cardiac magnetic resonance imaging to rule out structural abnormalities.

In cases refractory to medical therapy, interventional or surgical options may be considered. Percutaneous coronary intervention is generally reserved for short superficial bridges but carries high complication rates, including in-stent restenosis up to 75% with bare-metal stents and 25% with drug-eluting stents, as well as 6% stent perforation.^8^ Surgical management, such as myotomy (unroofing) or coronary artery bypass grafting (CABG), is indicated for deep (>5 mm) or long (>25 mm) bridges or when percutaneous coronary intervention fails.^9^ Myotomy directly relieves compression but may lead to chest pain recurrence in up to 60% of adults due to residual endothelial dysfunction.^8^ CABG using saphenous vein grafts has higher long-term patency (∼80%) compared to LIMA-LAD grafts (only 10% patent at 18 months) due to competitive flow. Outcomes data suggest lower major adverse cardiac event rates with myotomy (7%) than with CABG (41%).^8^ In patients presenting with ventricular tachyarrhythmias or cardiac arrest, implantable cardioverter-defibrillator placement is warranted for secondary prevention. After comparison, about 50% of patients were discharged with rate-controlling medications, and patients with an initial rhythm of ventricular fibrillation underwent implantable cardioverter-defibrillator placement or were recommended to receive one. Surgical unroofing was done in only 2 cases, and 1 patient refused surgical treatment. Overall, management should be tailored to anatomy, symptoms, and patient preferences, with a focus on hemodynamic control, risk factor modification, and cautious escalation to invasive interventions when medical therapy is insufficient.

## Conclusions

MB, although often considered benign, can have life-threatening consequences. Across published cases and our own, common features included ventricular fibrillation arrest, LAD involvement, and sudden collapse, often in otherwise healthy individuals. Recognition of MB's malignant potential is critical, particularly when patients present with ischemia or cardiac arrest in the absence of obstructive coronary artery disease. Although systolic bridging alone is frequently benign, tachycardia and other stressors that reduce diastolic perfusion can unmask its malignant potential. Early identification and appropriate secondary prevention strategies may improve outcomes in this under-recognized population.

## Funding Support and Author Disclosures

The authors have reported that they have no relationships relevant to the contents of this paper to disclose.

## Appendix

Video 1 Coronary Angiography Demonstrating Myocardial Bridging Coronary angiography demonstrating myocardial bridging in the mid- to distal left anterior descending artery (LAD) with associated coronary artery spasm, which improved after administration of intracoronary nitroglycerin. No significant coronary artery disease is noted in the LAD or diagonal branches. The constricted LAD portion is indicated by the red arrow in the video, which has been uploaded separately.

Video 2 Cardiac Arrest in 39-Year-Old Female With Myocardial Bridging.
